# Recurrent Stroke as a First Presentation in Behçet Disease: A Case Report

**DOI:** 10.7759/cureus.49222

**Published:** 2023-11-22

**Authors:** Salah Khafaji, Meshari S Alzahrani, Rabia Muddassir, Rakan A Almuhanna

**Affiliations:** 1 Neurology, Security Forces Hospital - Makkah, Makkah, SAU; 2 Internal Medicine, Security Forces Hospital - Makkah, Makkah, SAU

**Keywords:** neuro-behçet disease, vasculitis, brainstem lesion, recurrent stroke in young, behcet disease

## Abstract

Behçet disease (BD) is a multisystemic relapsing autoimmune vascular disorder. It is clinically characterized by recurrent oral ulcers, genital ulcers, eye, and skin manifestations. Development of neurological symptoms in BD cases is rare and occurs several years after the initial diagnosis. We describe a rare case of a 39-year-old Saudi male who presented with isolated neurological manifestations as the first sign of BD. The patient had recurrent strokes, both ischemic and hemorrhagic, over an 11-month period before developing typical BD features. A thorough investigation excluded other potential etiologies of his neurological disorders. Imaging showed multiple brainstem lesions compatible with parenchymal neuro-BD (NBD). The patient was positive for HLA-B51, a genetic marker linked to BD, but had a negative pathergy test. Treatment with corticosteroids and infliximab resulted in symptom improvement. The diagnosis of NBD requires a comprehensive clinical, imaging, and laboratory assessment to rule out other possible causes. This case demonstrates the need to include NBD in the differential diagnosis of young patients with unexplained neurological manifestations, especially if they are followed by an onset of BD features. Treatment with corticosteroids and biologic agents can achieve favorable outcomes. NBD can present with isolated neurological symptoms, emphasizing the need for a high level of suspicion and a multidisciplinary approach for accurate diagnosis and effective management.

## Introduction

Behçet disease (BD) is a multisystemic relapsing autoimmune vascular disorder. It is clinically characterized by recurrent oral ulcers, genital ulcers, and eye and skin manifestations [1].

The development of neurological symptoms occurs in less than 10% of BD cases and typically occurs several years after the initial diagnosis. It is quite variable in presentation and classified based on the localization of the affected lesion as parenchymal and nonparenchymal [2].

Here, we describe an unusual case of a male patient with isolated neurological symptoms as the initial presentation of neuro-Behçet disease (NBD) who subsequently developed the classical features of Behçet disease after 11 months of presentation.

## Case presentation

A 39-year-old Saudi male patient with a one-year history of type 2 diabetes mellitus on oral hypoglycemic agents and smoking one pack per day for the last ten years presented to the emergency department in September 2022 with left upper limb and right facial numbness for one day. He had a similar episode two months ago, lasting 30-60 minutes, that resolved spontaneously.

He had no medical history of hypertension or dyslipidemia. He had no family history of stroke or other neurological disorders. He had no history of stress or trauma. He denied any headache, visual loss, speech difficulty, weakness, or sensory loss in other parts of the body. He had no fever, chills, cough, chest pain, palpitations, dyspnea, abdominal pain, diarrhea, or urinary symptoms. He had no joint pain, skin rash, oral ulcers, or genital ulcers. He took his usual medications on the day of presentation and checked his blood glucose level, which was normal. He did not take any other medications or supplements. He had no drug or food allergies. He had no recent travel history or contact with sick people.

On general examination, his vital signs were blood pressure of 121/70 mmHg, pulse of 78 beats per minute, respiratory rate of 14 breaths per minute, temperature of 36.8°C, and oxygen saturation of 98% on room air. The patient was alert and oriented to time, place, and person. His systemic examination was unremarkable.

On neurological examination, higher mental function was intact. The cranial nerve exam was intact except for decreased sensation in the right face involving the V1, V2, and V3 branches of the trigeminal nerve. His motor exam showed slight pronator drift on the left side, and his tone was normal. His deep tendon reflexes were 2+ and symmetric. His sensation was intact to light touch and pinprick in the left upper extremity. He had normal plantar reflexes. His coordination was normal on both sides.

Upon arrival in the emergency department, a CT scan of his brain was ordered by the ER team, revealing no remarkable findings.

The patient was admitted as a case of suspected stroke in the young for workup. Basic laboratory tests, including complete blood count, electrolytes, renal function, liver function, and inflammatory markers, were normal. Electrocardiography showed normal sinus rhythm. His HbA1c was 8% (normal range: 4.80-5.90%), and lipid profile showed low-density lipoproteins 1.89 mmol/L (normal range: 0.50-4.00 mmol/L), high-density lipoproteins 0.84 mmol/L (normal range: >1.55 mmol/L), and triglyceride 0.86 mmol/L (normal range: 1.70-2.25 mmol/L).

Autoimmune workup, including antinuclear antibodies, anti-dsDNA antibodies, anticardiolipin antibodies, lupus anticoagulant, anti-beta2 glycoprotein I antibodies, C3 and C4, antineutrophil cytoplasmic antibodies, anti-Ro, anti-La, rheumatoid factor, and anti-cyclic citrullinated peptide, were all negative. Pathergy test was also negative.

Hypercoagulability workup, including factor V Leiden mutation, prothrombin gene mutation, protein C and S levels, antithrombin III level, homocysteine level, JAK2 V617F mutation, and JAK2 exons 12 and 13 mutations, were all unremarkable.

Infectious causes were ruled out by negative tests for hepatitis, syphilis, zoster, and human immunodeficiency virus. Cerebrospinal fluid analysis showed a total leucocyte count of 3 (normal range: 0-5 cells/uL), protein of 286 (normal range: 150-400 mg/L), and glucose of 3.5 (normal range: 2.2-3.9 mmo/L).

**Figure 1:**
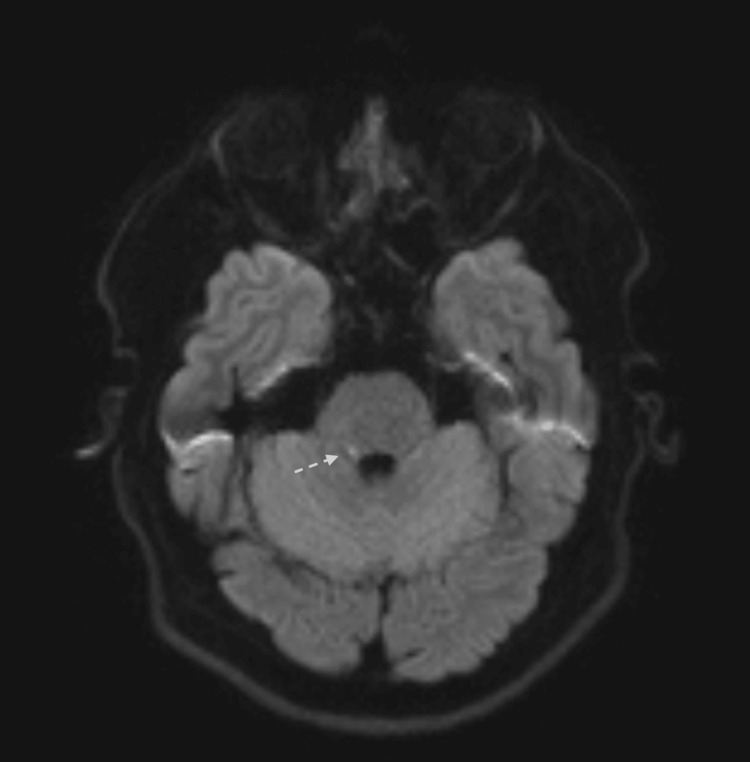
MRI brain DWI view showing small left pontine insult.

**Figure 2:**
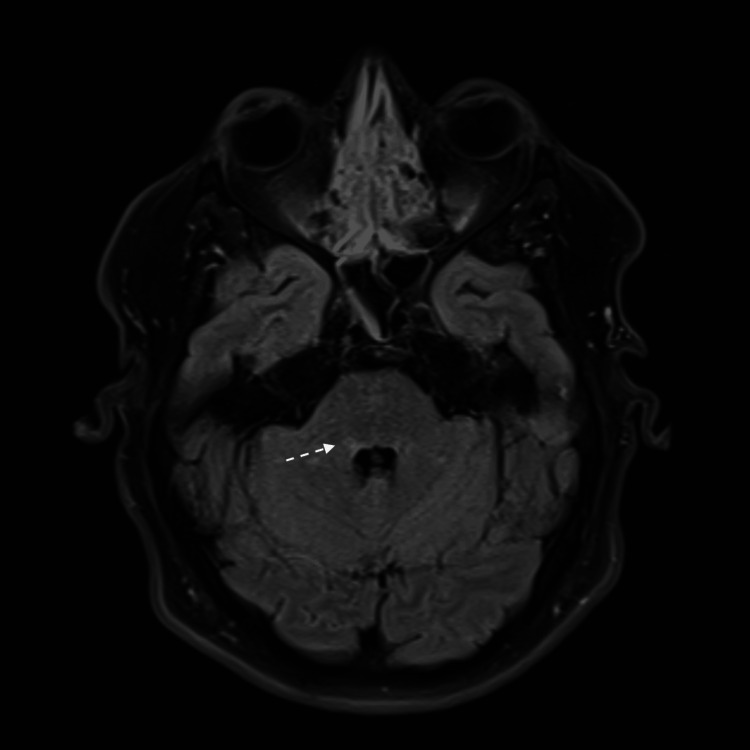
MRI brain FLAIR view showing small left pontine insult.

A limited magnetic resonance imaging (MRI) of the brain showed a small right pontine infarct that was hyperintense on fluid-attenuated inversion recovery (FLAIR) sequence and showed restricted diffusion on diffusion-weighted imaging (DWI) (Figures 1, 2). A computed tomography angiography (CTA) of the neck and intracranial vessels showed no evidence of stenosis, occlusion, dissection, or aneurysm. A transthoracic echocardiography (ECHO) showed normal cardiac chambers, valves, and function. No intracardiac shunt or thrombus was detected. A carotid and vertebral duplex ultrasound showed normal flow velocities and no plaque or stenosis. A 24-hour Holter study was normal.

The patient was discharged on antiplatelet medication, and the mechanism of the stroke was concluded to be lacunar etiology secondary to his heavy smoking and uncontrolled diabetes.

**Figure 3:**
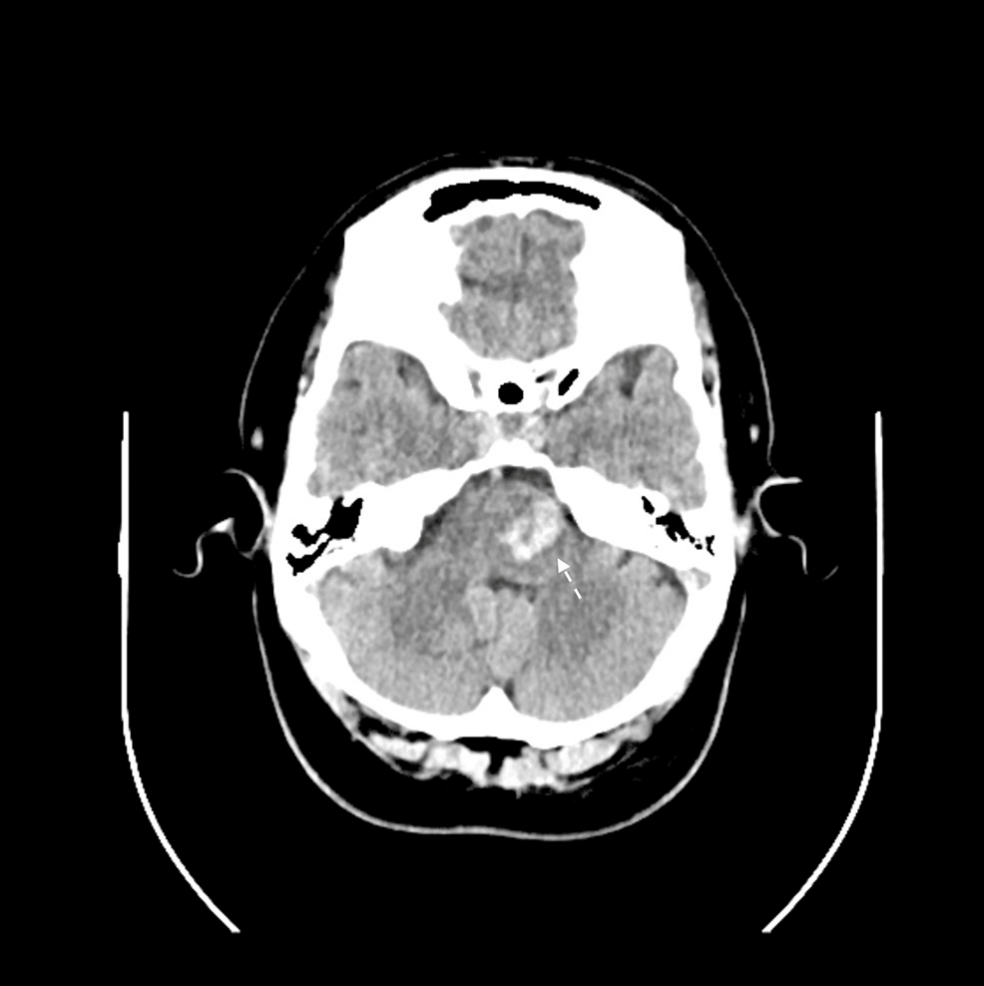
CT scan of the brain showing left pontine hemorrhage.

The patient presented to the emergency department in March 2023 with a new onset of left facial and right upper limb numbness. He had no other neurological symptoms. The examination was significant for decreased sensation in the left face and the right side of the body and positive right-side pronator drift. A non-contrast computed tomography (CT) scan of the brain revealed an acute left pontine hemorrhage measuring about 2.3 x 1.3 cm in size with minimal mass effect and no surrounding edema (Figure 3). The patient was admitted for a stroke workup.

**Figure 4:**
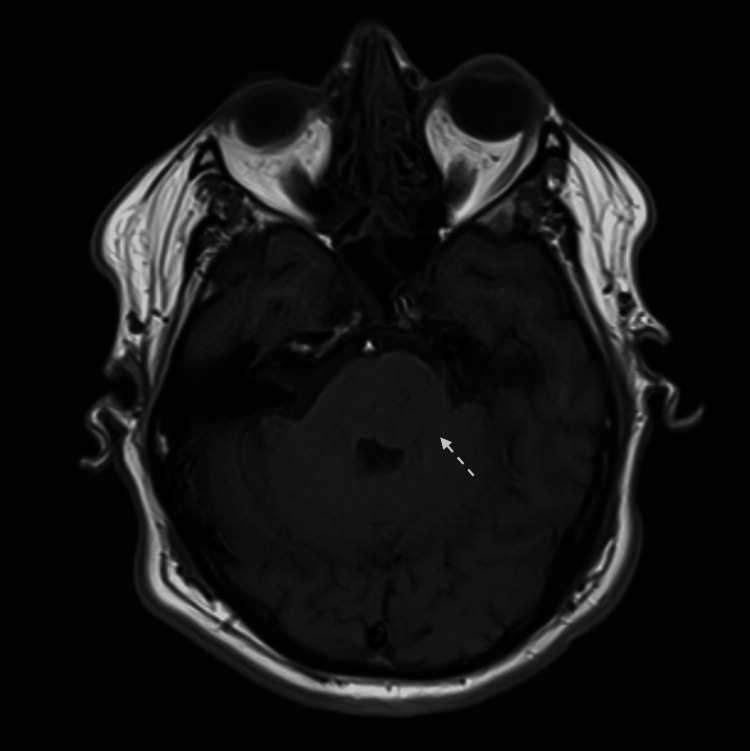
MRI brain T1 view showing asymmetry between right and left pons.

**Figure 5:**
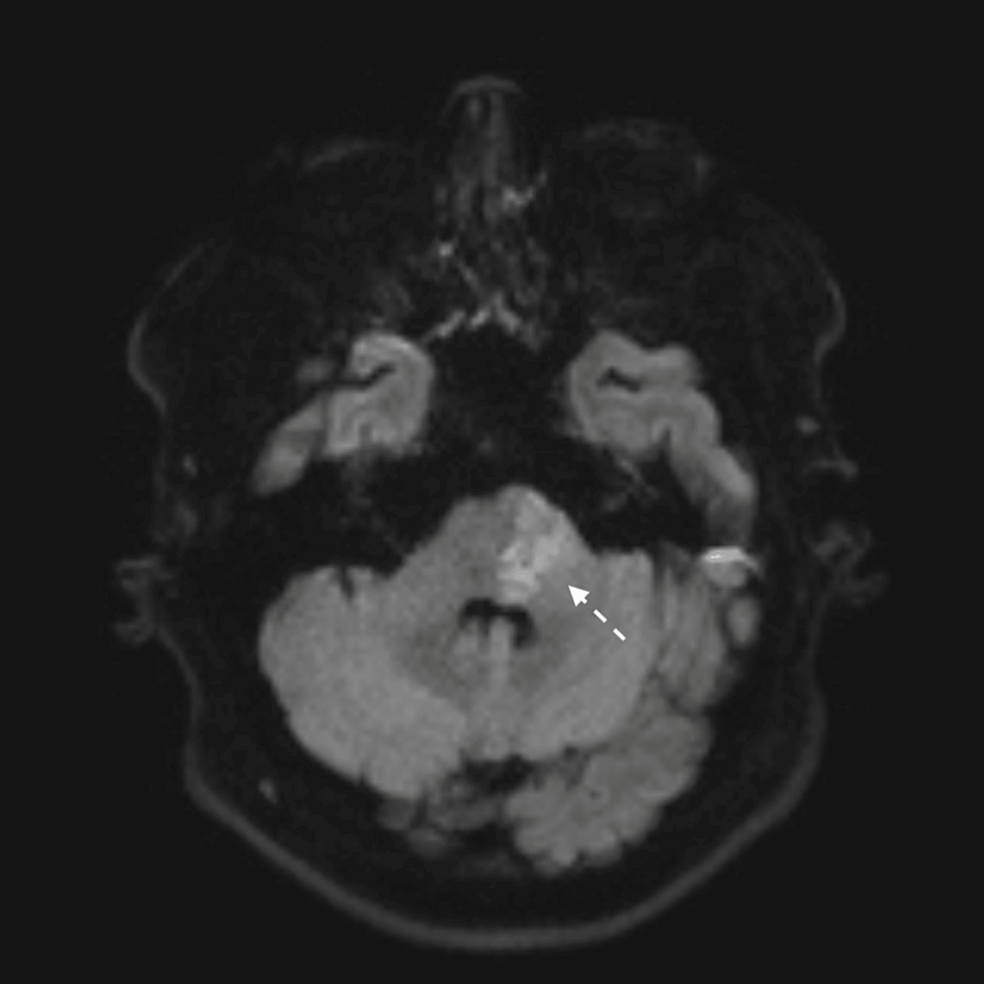
MRI brain DWI view showing left pontine diffusion restriction.

**Figure 6:**
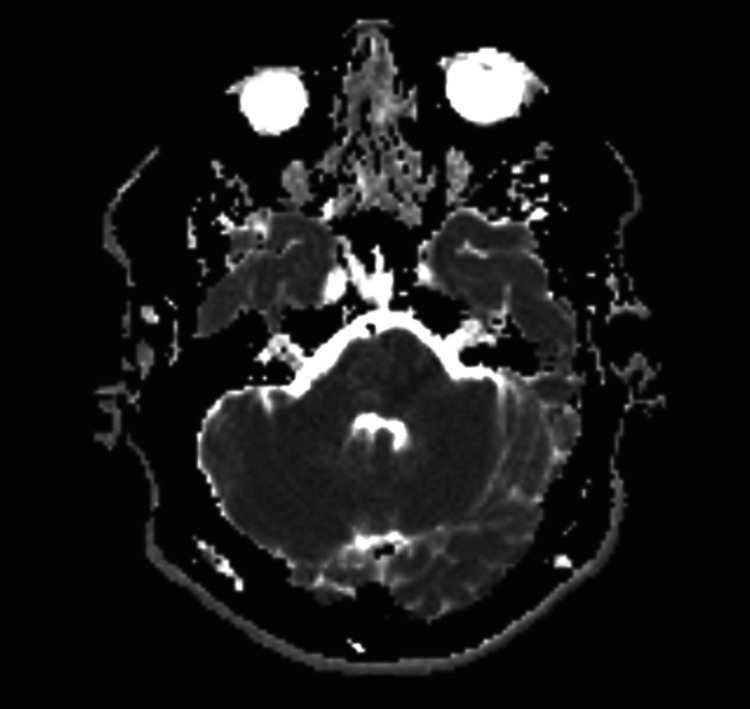
MRI brain ADC view showing left pontine low signal intensity diffusion restriction.

**Figure 7:**
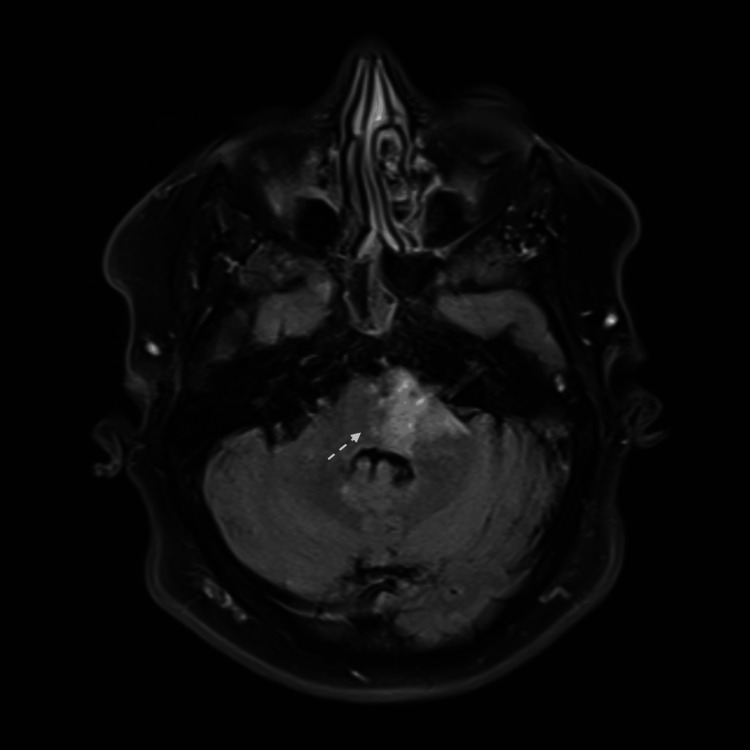
MRI brain FLAIR view showing left pontine hyperintense signal.

An MRI scan of the brain confirmed the presence of a new lesion in the left pons that was isointense on T1WI, hyperintense on T2WI, and FLAIR sequence and showed restricted diffusion on DWI (Figures 4-7). A magnetic resonance angiography (MRA) scan of the neck and intracranial vessels showed no evidence of stenosis, occlusion, dissection, or aneurysm.

As the patient was young with recurrent stroke, one ischemic and one hemorrhagic, raising the suspicion of vasculitis, he underwent vessel wall imaging and conventional angiography with repeated vasculitis workups, all of which were unremarkable.

Antiplatelet therapy was discontinued. The patient was discharged in stable condition. A follow-up CT brain scan was arranged after two weeks to decide about resuming antiplatelet therapy. The CT scan showed regression of the hemorrhage, and aspirin was resumed.

The patient presented to the ER in June 2023 with new onset left-sided facial numbness and right-sided numbness and weakness, along with a one-month history of recurrent oral and genital ulcers, arthralgia, and red eye.

On examination, neurological findings showed decreased sensation in the left face and right side of the body with a positive right-side pronator drift. Systemic examination was significant for painful oral ulcers, with three to four lesions described as round, well-circumscribed lesions with a red base.

Upon exposing the back of the patient, there were brownish follicular papules seen over the upper to mid portions of the back consistent with pseudofolliculitis. Upon examining the genitals of the patient, he had genital ulcers.

**Figure 8:**
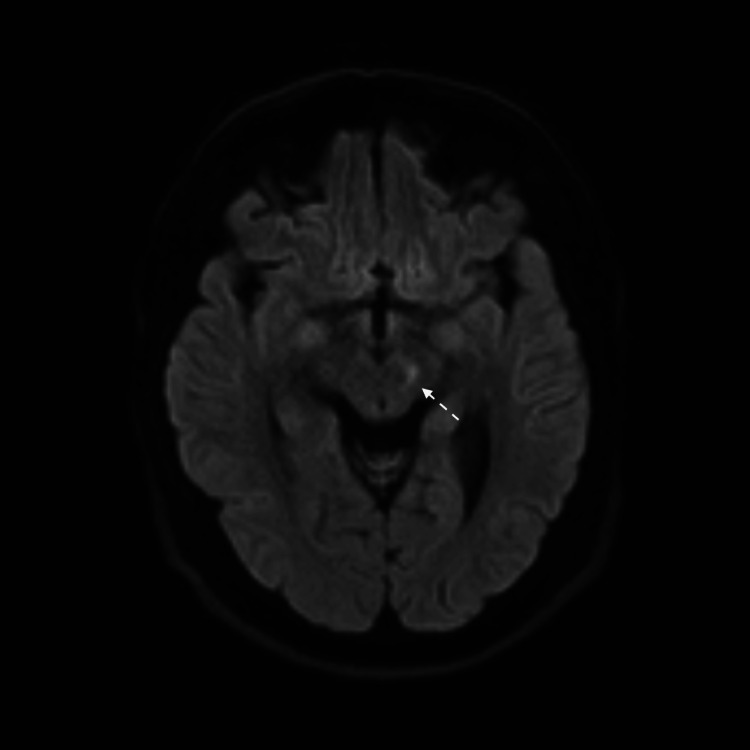
MRI brain DWI view shows left midbrain cerebral peduncle infarction.

**Figure 9:**
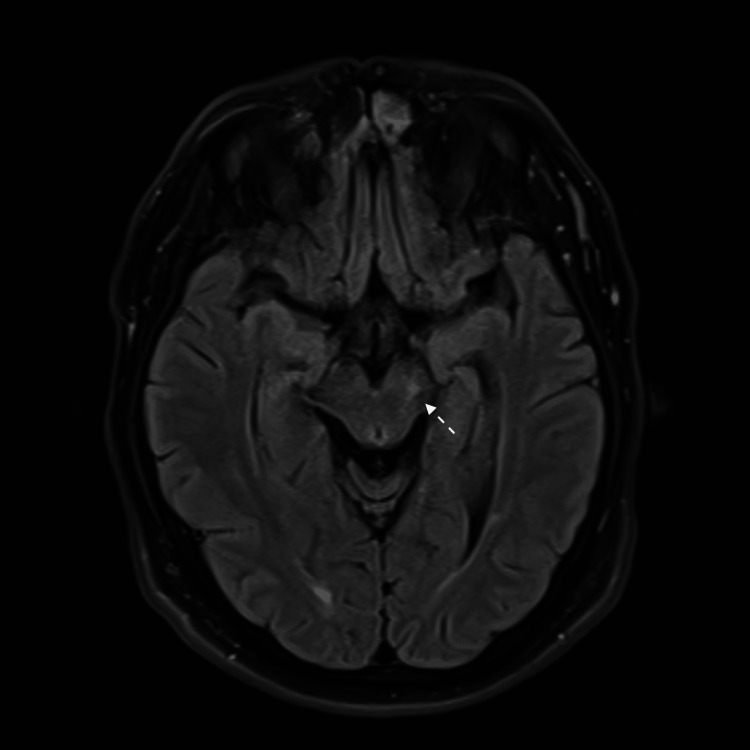
MRI brain FLAIR view shows left midbrain cerebral peduncle infarction.

An MRI brain revealed a tiny ischemic focus at the left cerebral peduncle of the midbrain, manifested by a bright signal on DWI and FLAIR sequences (Figures 8, 9). Laboratory workup was significant for positive HLA-B51. The repeated pathergy test was negative.

He was started on pulse steroids (methylprednisolone 1,000 mg intravenously for five days) followed by oral steroids. After consulting with the rheumatology department of our hospital, infliximab was added as maintenance therapy for the patient. His systemic symptoms improved upon follow-up.

## Discussion

BD is a chronic vascular inflammatory disease that occurs in young adults 20 to 40 years of age predominantly in males of the Arab population, and it is characterized by recurrent oral and genital ulcers, ocular lesions, and mucocutaneous manifestations [2]. NBD is a rare complication of BD seen in 3-9% of BD cases that involve the brain tissue or blood vessels in the brain, causing various neurological symptoms [1].

In one case series that gathered 200 patients of NBD, only six cases representing 3% of the sample size had the onset of neurological symptoms preceding the BD-characterized systemic symptoms. Interestingly three of the subjects had a one-year time gap before establishing the diagnosis of BD, and the remaining three had a time gap of two, five, and nine years [3].

We report an unusual case of a male patient with isolated neurological symptoms as the initial presentation of NBD who subsequently developed the classical features of BD after 11 months of presentation. The patient had recurrent episodes of stroke involving different parts of the brainstem, both ischemic and hemorrhagic, without any evidence of vascular stenosis, occlusion, dissection, or aneurysm on imaging studies. The patient also had positive HLA-B51, which is a genetic marker associated with BD, but a negative pathergy test, which is a nonspecific skin test for BD that involves injecting a sterile needle into the forearm and observing the reaction after 24-48 hours. The patient responded well to corticosteroids and infliximab, which is a biologic agent that inhibits tumor necrosis factor-alpha (TNF-α), a pro-inflammatory cytokine involved in the pathogenesis of BD.

The diagnosis of NBD is challenging as there is no specific diagnostic test or criteria for it [4]. The diagnosis is based on the clinical manifestations, imaging findings, laboratory tests, and exclusion of other causes of neurological disorders [4]. The differential diagnosis of NBD includes other causes of stroke in the young, such as vasculitis (e.g., systemic lupus erythematosus, antiphospholipid syndrome, granulomatosis with polyangiitis), thrombophilia (e.g., factor V Leiden mutation, prothrombin gene mutation), infection (e.g., syphilis, zoster), malignancy (e.g., lymphoma), and demyelination (e.g., multiple sclerosis) [5]. In our case, we ruled out these possibilities by performing an extensive workup for autoimmune, hypercoagulable, infectious, and neoplastic disorders.

The imaging findings of NBD are diverse and depend on the type and location of the lesion. Parenchymal NBD typically shows multiple lesions in the brainstem, basal ganglia, thalamus, internal capsule, and cerebral hemispheres that are hyperintense on T2-weighted images (T2WI) and fluid-attenuated inversion recovery (FLAIR) sequence and show restricted diffusion on diffusion-weighted imaging (DWI) [6]. Hemorrhagic lesions can also occur in parenchymal NBD because of necrosis and rupture of small vessels. Nonparenchymal NBD typically shows dural sinus thrombosis or arterial occlusion on MRA or conventional angiography. Vessel wall imaging can show thickening and enhancement of the vessel wall in NBD [6]. In our case, we observed multiple lesions in the brainstem that were consistent with parenchymal NBD. We also performed vessel wall imaging and conventional angiography to exclude nonparenchymal NBD.

The treatment options for NBD include corticosteroids, immunosuppressive agents (e.g., azathioprine, cyclophosphamide), and biologic agents (e.g., infliximab). Corticosteroids are usually given as pulse therapy followed by oral maintenance therapy. Immunosuppressive agents are added as steroid-sparing agents or for refractory cases. Biologic agents are used for severe or resistant cases or for patients who cannot tolerate immunosuppressive agents. In our case, we used corticosteroids and infliximab as maintenance therapy for the patient [1].

The prognosis and follow-up of NBD depend on the severity and frequency of relapses, involvement of other organs, and compliance with treatment [1]. The mortality rate of NBD was reported to be 9.8% for 42 out of 387 BD, depending on the type and location of the lesion [7]. The most common causes of death include brainstem involvement, intracranial hemorrhage, pulmonary artery aneurysm rupture, or infection [3,7,8]. The relapse rate of NBD is affected by the treatment regimen and duration, but there is a lack of specific data in the current literature. The relapse rate was estimated to be around 33% [4]. Positive HLA-B51 is a genetic marker that was found to be associated with an increased risk of relapse in NBD patients by 3.6-fold [9]. The factors associated with poor prognosis include male sex, young age, parenchymal involvement (especially brainstem lesions), relapse during steroid tapering, and progressive disease course [3,8]. In our case, the patient had a poor prognosis according to the literature review, as he was a male patient with multiple parenchymal relapses involving the brainstem before initiating treatment. One of these relapses resulted in a pontine hemorrhage. These factors indicate a poor clinical outcome for NBD. Additionally, the patient tested positive for HLA-B51. However, the patient showed a promising response to treatment. He was followed up regularly and advised to adhere to his medication regimen.

## Conclusions

In conclusion, we presented a rare case of NBD with isolated neurological symptoms as the initial presentation of BD. The diagnosis was based on the clinical manifestations, imaging findings, laboratory tests, and exclusion of other causes of neurological disorders. The patient responded well to corticosteroids and infliximab. The prognosis and follow-up of NBD depend on the severity and frequency of relapses, the involvement of other organs, and compliance with treatment. NBD is a challenging diagnosis that requires a high index of suspicion and a multidisciplinary approach.
